# Application of omnibearing operating room nursing in total hip arthroplasty and its influence on postoperative pain

**DOI:** 10.1590/1414-431X2025e14743

**Published:** 2026-01-09

**Authors:** Lei Lu, Jian Shen, Haiwei Zhang

**Affiliations:** 1 Department of Anesthesiology and Surgery, The First Affiliated Hospital of Nanjing Medical University, Nanjing, Jiangsu Province, China

**Keywords:** Total hip arthroplasty, Omnibearing operating room care, Postoperative pain, Hip function, Complications, Quality of life

## Abstract

We aimed to explore the effect of omnibearing operating room nursing in total hip arthroplasty (THA) and its influence on postoperative pain. One hundred and twenty patients who underwent THA were enrolled and assigned into either a control group or an observation group, with 60 cases in each group. The control group received routine nursing, and the observation group received omnibearing operating room nursing in addition to routine nursing. Perioperative indicators, postoperative pain, physical signs, hip function, quality of life, complications, as well as nursing satisfaction were compared between the two groups. Compared to the control group, the observation group showed shorter operation time and hospital stay, less intraoperative blood loss, lower visual analogue scale (VAS) scores post-operation, a lower rate of additional analgesic use, and decreased respiratory rate, systolic blood pressure (SBP), and diastolic blood pressure (DBP) at 7 days after operation, as well as elevated Harris Hip Score (HHS) dimension scores, total scores, and SF-36 scores, lower total incidence rate of complications, and higher total satisfaction rate of nursing care (all P<0.05). Omnibearing operating room nursing can relieve postoperative pain, promote the recovery of hip joint function, improve the quality of life, decrease the occurrence of complications, and enhance patients' nursing satisfaction.

## Introduction

Total hip arthroplasty (THA) is recognized as one of the most successful and widely performed surgical procedures in orthopedic medicine (1). Although THA has become a standard procedure adeptly managed by orthopedic surgeons, certain cases can still present complexities. These challenges may arise from coexisting health issues such as obesity, dermatological conditions, muscular disorders, a history of neurological illnesses, or related skeletal deformities (2). Effective pain management is crucial, as it can lower the overall direct medical expenses associated with lower extremity joint replacement surgeries by minimizing hospital stays and the resources required during recovery. Factors that contribute to shorter hospital durations include consistent staffing and continuity in nursing care, timely and relevant information regarding short stays, functional discharge criteria, early mobilization, and a multimodal analgesic strategy that emphasizes reducing opioid use (3).

Maintaining a high standard of nursing care is critical throughout the entire patient period, from the initial consideration of THA or total knee replacement (TKR) to the moment the patient is brought into the operating room. Nurse practitioners in primary care are ideally situated to conduct a comprehensive assessment of patients, determining the necessity for surgery and ensuring that patients are physically, psychologically, and socially prepared before referring them to an orthopedic surgeon (3). Historically, operating room (OR) nursing referred to the care provided to patients during the surgical phase and the support offered within the OR environment (4). Today, OR nursing is essential for facilitating efficient surgical processes and enhancing surgical outcomes. Evidence-based nursing (EBN) has increasingly supplanted traditional nursing practices, as it is specifically designed to address the individualized nursing needs of patients (5). Nursing care provided in the operating room for patients undergoing THA can help stabilize postoperative life indicators, enhance recovery after surgery, decrease the occurrence of negative surgical outcomes, and lead to improved evaluations (6). According to Lucchini et al., implementing standardized nursing interventions in the OR can significantly enhance the clinical outcomes of THA (7). To sum up, this study aimed to investigate the impact of omnibearing operating room nursing on postoperative recovery of patients undergoing THA, ultimately providing evidence to evaluate the effectiveness of omnibearing operating room nursing in THA and its impact on postoperative pain.

## Material and Methods

### Participants

The study population was patients who received THA in the Jiangsu Province Hospital, China, from January 2023 to July 2024.

Inclusion criteria for the study included the following: patients who meet the surgical indications by CT examination, patients undergoing the first THA, patients with stable vital signs, and patients with complete information.

The exclusion criteria included the following: patients with congenital hip joint deformity or severe osteoporosis, patients who cannot tolerate surgical treatment and cannot cooperate with this study, patients with organ parenchymal diseases, patients with malignant tumors, patients with coagulation disorders, patients with infectious diseases, patients with mental disorders, and patients who withdraw from the study halfway.

After screening a total of 161 patients by the inclusion and exclusion criteria, 120 patients were eligible and were allocated into the control group and the observation group following different nursing protocols, with 60 cases in each group. In the control group, there were 34 males and 26 females, aged 48-73 years, with a mean age of 58.57±6.98 years. There were 27 cases of hip arthritis, 18 cases of femoral head necrosis, and 15 cases of femoral neck fracture. In the observation group, there were 36 males and 24 females, aged 47-71 years, with a mean age of 59.15±5.98 years. There were 24 cases of hip arthritis, 20 cases of femoral head necrosis, and 16 cases of femoral neck fracture. The two groups were balanced and comparable in general data (P>0.05).

This study was reviewed and approved by the Ethics Committee of Jiangsu Province Hospital. Written informed consent was acquired from all participants.

### Nursing intervention

The control group was given routine nursing. Before surgery, the nursing staff assessed the patient's condition, carried out routine examinations, and made preoperative preparations. During the operation, the nursing staff inspected the disinfection of surgical instruments, maintained the aseptic state of the surgical environment, assisted the surgeon to complete the surgical operation, and closely monitored various vital signs of the patient. If the patient had abnormalities, nurses immediately informed the doctor and performed the corresponding treatment. After surgery, the nursing staff sent the patient to the ward after the vital signs were stable, developed a rehabilitation plan according to the patient's rehabilitation process, and assisted the patient in postoperative functional exercise.

The observation group was given omnibearing operating room nursing in addition to routine nursing. The specific methods were as follows: 1) An omnibearing operating room nursing group was established. The members of the group were composed of senior operating room nurses, anesthesiologists, and postoperative rehabilitation nursing experts with extensive clinical experience and professional training. 2) Preoperative nursing: the nursing staff provided popular information about the surgery for the patients and their families, informed surgical precautions, and addressed the patients' questions, helping them develop a correct understanding of both the risk and the safety of surgery. The nursing staff evaluated the psychological status of the patients and developed the corresponding psychological nursing plan for them according to the evaluation results. The nursing staff timely relieved the negative emotions of patients, guided family members to encourage patients, cheered patients by describing successful cases, increased patients' confidence in surgery, and used animation, video, manual and other means to reduce the anxiety and fear related to the surgery. 3) Intraoperative nursing: the nursing staff ensured gentle and safe movement and reduced unnecessary movement and postural changes when transporting the patient. The nursing staff adjusted the operating room temperature to 24∼26°C, and maintained the humidity at 50∼60%, so as to create the optimal operating environment for the patient. The nursing staff minimized skin exposure of the patient's non-surgical site and kept the patient warm during surgical procedures with blankets and cotton foot covers. The nursing staff strictly inspected all articles and related equipment that could be used during the operation and sorted out the surgery-related supplies in an orderly manner according to the surgical operation process. The nursing staff monitored the vital signs of the patient every 15 min, and recorded the blood pressure, heart rate, respiratory rate, and body temperature of the patient in real time through the central monitoring system. The nursing staff ensured that an #18 vein indwelling needle was used to puncture the patient, ensured the shortest opening time of the infusion channel, regularly checked whether the infusion channel of the patient was unobstructed, and observed whether there was leakage at the infusion site. The nursing staff assisted the anesthesiologist in performing an effective anesthesia, assisted the physician in wound cleaning and drainage, and timely solved the problems that arose during the operation. During the procedure, the nursing staff placed the family member in a comfortable waiting area to wait for the procedure to be completed. 4) Postoperative nursing: when the operation was completed, the nursing staff transferred the patient to the postoperative recovery room and prepared a written description of the operation process. They placed pads between the patient's skin and the pelvic holder and provided gradient compression stockings to the patient. The nursing staff continued to closely monitor the patient's vital signs and gave the patient anticoagulant management and pain management. The nursing staff closely observed the patient's wound healing, regularly changed sterile dressings, kept the incision clean and dry, and protected the wound from moisture and dirt. The nursing staff instructed the patient to perform active and passive rehabilitation exercises of the lower limbs to promote the recovery of hip joint function.

### Perioperative indicators

Perioperative indicators, including operation time, intraoperative blood loss, and hospital stay, were compared between the two groups.

### Postoperative pain

Visual analogue scale (VAS) (8) was used to assess pain level in the two groups at 24, 48, and 72 h after surgery. Patients were instructed to rate their pain on a scale from 0 to 10, with 0 indicating no pain and 10 indicating unbearable pain. Postoperative use of additional analgesic medication was documented.

### Indicators of physical signs

Respiratory rate, systolic blood pressure (SBP), and diastolic blood pressure (DBP) were monitored by ECG pre-operation and 7 days post-operation.

### Hip function

Pre-operation and 7 days post-operation, the Harris Hip Score (HHS) (9) was used to assess hip function in the two groups, including four dimensions: pain (44 points), function (47 points), joint range of motion (5 points), and deformity (4 points), with a total score of 100 points and higher scores indicating better function recovered.

### Quality of life

Quality of life in both groups was assessed by the Health Status Questionnaire (SF-36) (10) pre-operation and 7 days post-operation, which has 36 items and a total score of 100 points. Higher scores indicated better quality of life of patients.

### Complications

At 7 days post-operation, postoperative complications such as joint dislocation, incision infection, and prosthesis loosening were observed in the two groups.

### Satisfaction with nursing

Before patients were discharged from the hospital, satisfaction with nursing was measured in both groups. The patients were given a nursing satisfaction questionnaire developed by the hospital, which included basic nursing, nursing techniques, and nursing attitude, with a total score of 100 points, which were divided into satisfactory (≥80 points), basically satisfactory (61∼79 points), and unsatisfactory (≤60 points), and satisfaction rate was calculated as satisfaction = (satisfactory + basically satisfactory) / total number of cases × 100%.

### Statistical analysis

Data analysis was performed using SPSS 26.0 (IBM Corp., USA) and GraphPad Prism 8.0.2 software (Graph Pad Inc., USA). Enumeration data are reported as percentages (%) and compared using the χ^2^ test. Measurement data are reported as means±SD, and paired sample *t*-test was utilized for intra-group comparison and independent sample *t*-test was adopted for inter-group comparison. Differences were considered significant at P<0.05.

## Results

### Perioperative indicators

The operation time and hospital stay in the observation group were shorter and intraoperative blood loss was lower compared to the control group (P<0.05) (Table 1). The results showed that omnibearing operating room nursing shortened the operation time and hospital stay of patients with THA, and reduced intraoperative blood loss.

**Table 1:** Comparison of perioperative indicators between patients treated with omnibearing operating room nursing and control patients.

Perioperative indicators	Control group (n=60)	Observation group (n=60)	*t* value	P value
Operative time (min)	82.85±7.47	70.82±8.96	7.994	<0.001
Intraoperative blood loss (mL)	312.40±36.76	247.80±28.88	10.704	<0.001
Length of stay (d)	9.23±1.62	5.37±1.19	14.892	<0.001

Data are reported as mean and SD. Student's *t*-test.

### Postoperative pain

Lower VAS scores were noted at 24, 48, and 72 h after operation in the observation group in contrast to the control group (P<0.05). The usage rate of additional postoperative analgesic drugs in the observation group was lower than that in the control group (P<0.05) (Table 2). These results revealed that omnibearing operating room nursing could relieve postoperative pain in patients undergoing THA.

**Table 2:** Comparison of postoperative pain between patients treated with omnibearing operating room nursing and control patients in total hip arthroplasty.

Postoperative pain	Control group (n=60)	Observation group (n=60)	*t*/χ^2^ value	P value
VAS score				
24 h after operation (points)	6.27±1.27	4.95±0.75	6.909	<0.001
48 h after operation (points)	4.05±0.67	2.33±0.48	16.113	<0.001
72 h after operation (points)	2.48±0.50	1.42±0.50	11.672	<0.001
Usage rate of additional analgesic drugs	18 (30.00%)	4 (6.67%)	9.755	0.002

Data are reported as mean and SD and n (%). Student's *t*-test and chi-squared test. VAS: visual analogue scale.

### Physical indicators

Before operation, there was no significant difference in respiratory rate, SBP, and DBP between the two groups (P>0.05). At 7 days after operation, the respiratory rate, SBP, and DBP in the two groups were markedly higher than those before operation, but were lower in the observation group compared to the control group (P<0.05) (Table 3). It was suggested that omnibearing operating room nursing was beneficial to improve the respiratory rate and blood pressure level of patients undergoing THA.

**Table 3:** Comparison of respiratory rate, SBP, and DBP between patients treated with omnibearing operating room nursing and control patients.

Physical indicators	Control group (n=60)	Observation group (n=60)	*t* value	P value
Respiratory rate (breaths/min)				
Pre-operation	16.98±1.50	17.07±1.43	0.312	0.756
7 days post-operation	20.18±1.91*	18.40±1.42*	5.811	<0.001
SBP (mmHg)				
Pre-operation	121.83±9.03	121.22±9.10	0.373	0.710
7 days post-operation	134.87±6.96*	126.48±7.17*	6.495	<0.001
DBP (mmHg)				
Pre-operation	78.30±6.69	78.62±8.20	0.232	0.817
7 days post-operation	86.75±8.63*	80.17±11.16*	3.615	<0.001

Data are reported as mean and SD. *P<0.05 compared with the same group before operation (Student's *t*-test). SBP: systolic blood pressure; DBP: diastolic blood pressure.

### Hip function

Before operation, there was no notable difference in HHS dimension scores and total scores between the two groups (P>0.05). At 7 days after operation, the scores of HHS dimension and total score in the two groups were markedly higher than those before operation, and there was a significant elevation in HHS dimension scores and total score in the observation group compared to the control group (P<0.05) (Table 4). To conclude, omnibearing operating room nursing could promote the recovery of hip joint function in patients undergoing THA.

**Table 4:** Comparison of dimensions and total HHS score between patients treated with omnibearing operating room nursing and control patients.

HHS score	Control group (n=60)	Observation group (n=60)	*t* value	P value
Pain (points)				
Pre-operation	26.17±7.15	25.83±6.46	0.268	0.789
7 days post-operation	33.13±4.88*	39.47±4.07*	7.728	<0.001
Function (min)				
Pre-operation	26.43±3.53	27.05±3.21	1.001	0.319
7 days post-operation	33.77±3.13*	40.10±2.77*	11.752	<0.001
Range of motion (points)				
Pre-operation	2.03±0.18	1.98±0.13	1.742	0.084
7 days post-operation	3.38±0.49*	4.03±0.41*	7.875	<0.001
Deformity (points)				
Pre-operation	1.73±0.45	1.70±0.46	0.402	0.688
7 days post-operation	2.38±0.49*	3.28±0.45*	10.428	<0.001
Total score (points)				
Pre-operation	56.37±10.81	56.57±9.71	0.107	0.915
7 days post-operation	72.67±8.47*	86.88±6.87*	10.103	<0.001

Data are reported as mean and SD. *P<0.05 compared with the same group before operation (Student's *t*-test). HHS: Harris Hip Score.

### Quality of life

Before surgery, the difference in SF-36 scores between the two groups had no statistical significance (P>0.05). At 7 days after operation, SF-36 scores in both groups were sharply higher than those before operation, and the observation group exhibited increased SF-36 scores compared to the control group (P<0.05) (Table 5). These findings disclosed that omnibearing operating room nursing could improve the quality of life of patients with THA.

**Table 5:** Comparison of SF-36 score between patients treated with omnibearing operating room nursing and control patients.

SF-36 score (points)	Control group (n=60)	Observation group (n=60)	*t* value	P value
Pre-operation	65.83±7.92	66.30±8.02	0.321	0.749
7 days post-operation	74.28±8.24*	83.45±7.36*	6.424	<0.001

Data are reported as mean and SD. *P<0.05, compared with the same group before operation (Student's *t*-test). SF-36: Health Status Questionnaire.

### Incidence of complications

The total incidence rate of complications in the observation group was notably lower relative to the control group (P<0.05) (Table 6). Omnibearing operating room nursing reduced the complications of THA patients.

**Table 6:** Comparison of incidence of complications between patients treated with omnibearing operating room nursing and control patients.

Complications	Control group (n=60)	Observation group (n=60)	χ^2^ value	P value
Joint dislocation	3 (5.00%)	1 (1.67%)	-	-
Prosthetic loosening	2 (3.33%)	0 (0.00%)	-	-
Incision infection	3 (5.00%)	0 (0.00%)	-	-
Total occurrence	8 (13.33%)	1 (1.67%)	5.886	0.015

Data are reported as n (%). Chi-squared test.

### Nursing satisfaction

The total satisfaction rate of nursing care in the observation group was remarkably higher in comparison to the control group (P<0.05) (Table 7). The results reflected that omnibearing operating room nursing could improve the nursing satisfaction of patients with THA.

**Table 7:** Comparison of nursing satisfaction between patients treated with omnibearing operating room nursing and control patients.

Satisfaction	Control group (n=60)	Observation group (n=60)	χ^2^ value	P value
Satisfactory	29 (48.33%)	37 (61.67%)	-	-
Basically satisfactory	18 (30.00%)	20 (33.33%)	-	-
Unsatisfactory	13 (21.67%)	3 (5.00%)	-	-
Total satisfaction	47 (78.33%)	57 (95.00%)	7.212	0.007

Data are reported as n (%). Chi-squared test.

## Discussion

THA is the recommended approach for patients with advanced hip arthritis resulting from developmental hip dislocation or dysplasia (11). As THA is an implantable surgery, it is associated with significant trauma requiring prolonged bed rest for patients postoperatively. This situation increases the likelihood of various complications. However, high-quality nursing care can help decrease the risk of these complications and facilitate patient recovery (12).

Clinical nursing pathway involves creating a nursing process based on the patterns of disease occurrence and progression, implementing nursing interventions in strict adherence to this pathway to enhance standardization. In developing these pathways, principles of evidence-based medicine are applied, ensuring that diseases are managed consistently. This approach helps standardize medical practices, minimize variations, reduce costs, and enhance the quality of care (12). This study aimed to investigate the application of omnibearing operating room nursing in THA and its impact on postoperative pain. Ultimately, the study sought to provide evidence-based recommendations to enhance nursing practices and improve patient outcomes after THA.

At first, our results showed that omnibearing operating room nursing can relieve postoperative pain in patients undergoing THA, which is consistent with the findings of the study by Liu et al. (13). In their study, Liu et al. demonstrated that the duration of hospital stay, weight-bearing period, and fracture recovery time are notably reduced in the study group (operating room nursing based on clinical quantitative assessment plus WeChat health education) compared to the control group (routine intervention). On the second and third days following surgery, the VAS scores of the patients treated with a combination of operating room nursing on the basis of clinical quantitative assessment and WeChat health education were considerably lower (13). In addition, our study suggested that the usage rate of postoperative analgesic drugs in the observation group was significantly lower than that in the control group, which further demonstrates the beneficial effect of omni-directional operating room nursing in improving pain status among patients undergoing THA. This may be attributed to effective pain relief, which reduced the need for postoperative analgesic medication in these patients.

Omnibearing operating room nursing was also beneficial in improving respiratory rate and blood pressure in patients undergoing THA. Our findings are in accordance with a previous study that demonstrated that patients treated with evidence-based nursing demonstrated marked improvements in blood pressure and heart rate following care, as well as notable reductions in their Self-Rating Anxiety Scale and Self-Rating Depression Scale scores (5). Additionally, we found that omnibearing operating room nursing reduced the complications of THA patients, which is consistent with the findings of Huang et al. (12). The use of clinical nursing pathways in postoperative care for THA or TKR can significantly lower the rates of complications and wound infections, as well as alleviate wound pain. Additionally, this approach enhances patient satisfaction with treatment, allowing for earlier hospital discharge.

Finally, we observed that omnibearing operating room nursing improved the quality of life and the nursing satisfaction of patients with THA, which is consistent with the results of Yu et al. (5). By alleviating patients' negative emotions and discomfort, nursing satisfaction and quality of life of patients were significantly higher in the patients treated with evidence-based nursing, as indicated by the nursing satisfaction survey. The implementation of evidence-based nursing in the operating room can enhance patient satisfaction with nursing care and improve their quality of life.

In summary, the application of omnibearing operating room nursing in THA reduced postoperative pain, enhanced recovery, and improved overall patient satisfaction. By focusing on comprehensive and coordinated care, this approach leads to better clinical outcomes and a more positive surgical experience for patients.

## Data Availability

The datasets generated and/or analyzed during the current study are available from the corresponding author on reasonable request.

## References

[1] Markatos K, Savvidou OD, Foteinou A, Kosmadaki S, Trikoupis I, Goumenos SD (2020). Hallmarks in the history and development of total hip arthroplasty. Surg Innov.

[2] Boisgard S, Descamps S, Bouillet B (2013). Complex primary total hip arthroplasty. Orthop Traumatol Surg Res.

[3] Young AC, Buvanendran A (2014). Pain management for total hip arthroplasty. J Surg Orthop Adv.

[4] Niu L, Li HY, Tang W, Gong S, Zhang LJ (2017). Evolving safety practices in the setting of modern complex operating room: role of nurses. J Biol Regul Homeost Agents.

[5] Yu X, Hu D, Chen L (2022). Evidence-based nursing in the operating room of obstetrics and gynecology departments alleviates patients' adverse moods and improves their quality of life. Am J Transl Res.

[6] Xu H, Yao X (2023). Analysis of the application value of nursing cooperation in operating room in total hip arthroplasty. Minerva Surg.

[7] Lucchini S, Castagnini F, Giardina F, Tentoni F, Masetti C, Tassinari E (2021). Cementless ceramic-on-ceramic total hip arthroplasty in post-traumatic osteoarthritis after acetabular fracture: long-term results. Arch Orthop Trauma Surg.

[8] Zhao X, Bai R, Yang J (2022). Effect of painless rehabilitation nursing for hip replacement patients. Comput Math Methods Med.

[9] Gotz JS, Leiss F, Maderbacher G, Meyer M, Reinhard J, Zeman F (2022). Implementing fast-track in total hip arthroplasty: rapid mobilization with low need for pain medication and low pain values: retrospective analysis of 102 consecutive patients. Z Rheumatol.

[10] Barone G, Zinno R, Pinelli E, Pair Study G, Benvenuti F, Bragonzoni L (2021). Evaluation of the efficacy and safety of an exercise program for persons with total hip or total knee replacement: study protocol for a randomized controlled trial. Int J Environ Res Public Health.

[11] Tözün IR, Beksaç B, Sener N (2007). Total hip arthroplasty in the treatment of developmental dysplasia of the hip [in Tukish]. Acta Orthop Traumatol Turc.

[12] Huang Z, Li Y, Peng J, Wang H, Shen K, Li Y (2024). Effects of clinical nursing pathway on the surgical site wound infection in patients undergoing knee or hip replacement surgery: a meta-analysis. Int Wound J.

[13] Liu Q, Wang J, Han J, Zhang D (2022). Effect of combining operating room nursing based on clinical quantitative assessment with WeChat health education on postoperative complications and quality of life of femoral fracture patients undergoing internal fixation. J Healthc Eng.

